# Urm1: A Non-Canonical UBL

**DOI:** 10.3390/biom11020139

**Published:** 2021-01-22

**Authors:** Martin Termathe, Sebastian A. Leidel

**Affiliations:** 1 Institute of Biochemistry, Protein Biochemistry and Photobiocatalysis, University of Greifswald, Felix-Hausdorff-Strasse 4, 17489 Greifswald, Germany; martin.termathe@uni-greifswald.de; 2 Department of Chemistry and Biochemistry, University of Bern, Freiestrasse 3, 3012 Bern, Switzerland

**Keywords:** 2-thiolation, tRNA modification, thiocarboxylate, rhodanese, sulfur-carrier protein, non-canonical UBL, ubiquitin-like protein

## Abstract

Urm1 (ubiquitin related modifier 1) is a molecular fossil in the class of ubiquitin-like proteins (UBLs). It encompasses characteristics of classical UBLs, such as ubiquitin or SUMO (small ubiquitin-related modifier), but also of bacterial sulfur-carrier proteins (SCP). Since its main function is to modify tRNA, Urm1 acts in a non-canonical manner. Uba4, the activating enzyme of Urm1, contains two domains: a classical E1-like domain (AD), which activates Urm1, and a rhodanese homology domain (RHD). This sulfurtransferase domain catalyzes the formation of a C-terminal thiocarboxylate on Urm1. Thiocarboxylated Urm1 is the sulfur donor for 5-methoxycarbonylmethyl-2-thiouridine (mcm^5^s^2^U), a chemical nucleotide modification at the wobble position in tRNA. This thio-modification is conserved in all domains of life and optimizes translation. The absence of Urm1 increases stress sensitivity in yeast triggered by defects in protein homeostasis, a hallmark of neurological defects in higher organisms. In contrast, elevated levels of tRNA modifying enzymes promote the appearance of certain types of cancer and the formation of metastasis. Here, we summarize recent findings on the unique features that place Urm1 at the intersection of UBL and SCP and make Urm1 an excellent model for studying the evolution of protein conjugation and sulfur-carrier systems.

## 1. Introduction

Urm1 (ubiquitin related modifier 1) is a non-canonical ubiquitin-like protein (UBL). Classical UBLs, like ubiquitin, SUMO (small ubiquitin-related modifier) or Nedd8, are conjugated to target proteins following an intricate enzymatic cascade. First, UBLs are adenylated at their conserved C-terminal diglycine motif by a dedicated activating enzyme (E1). Subsequently, a transthioesterification reaction with their conjugating enzyme (E2) occurs. Finally, an E3 ligase catalyzes the formation of an isopeptide bond between the C-terminus of the UBL and a lysine side chain of a target protein [1]. This protein conjugation can trigger different downstream processes depending on the type of UBL or whether it forms chains with specific linkages. UBL attachment can mark proteins for proteasomal degradation, but can also alter their localization or activity.

**Figure 1:**
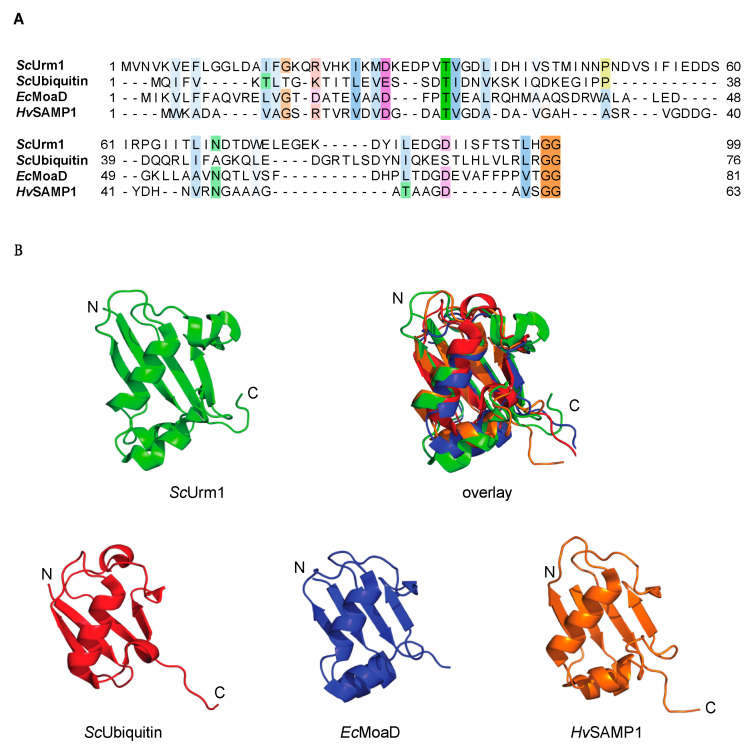
Comparison of Urm1, ubiquitin, SAMP1, and MoaD. (**A**) Structure-based sequence alignment of Urm1 (UniProt entry P40554), ubiquitin (UniProt entry P0CH08) from *Saccharomyces cerevisiae*, SAMP1 (UniProt entry D4GUF6) from *Haloferax volcanii* and MoaD (UniProt entry P30748) from *Escherichia coli.* The degree of conservation is reflected by color intensity. Sequences were aligned with PROMALS3D [5] and visualized using Jalview [6]. (**B**) Richardson ribbon diagrams of the three-dimensional schematic representation of protein structures of *Sc*Urm1 (PDB entry 2QJL), *Sc*Ubiquitin (PDB entry 3CMM), *Ec*MoaD (PDB entry 1JW), and *Hv*SAMP1 (PDB entry 3PO0). Structures were superimposed and visualized using PyMol [7] and their N- and C-terminus are indicated.

Urm1 was first identified in a homology search in the *Saccharomyces cerevisiae* genome [2]. The goal of the study was to identify unknown UBLs distantly related to ubiquitin. Therefore, the query did not use known UBL. Bacterial sulfur-carrier protein (SCP) systems share an identical activating mechanism with UBLs. The bacterial MoaD (molybdopterin synthase sulfur carrier subunit) and ThiS (thiamin biosynthesis protein) were chosen in the query to search for distantly related ubiquitin-like conjugation systems [1,2]. The MoaD-MoeB and ThiS-ThiF systems provide sulfur for the biosynthesis of the cofactors molybdopterin and thiamin, respectively. In brief, MoaD and ThiS are adenylated by their cognate E1-like activating enzyme MoeB (molybdopterin-synthase sulfurylase) and ThiF and subsequently thiocarboxylated at their C-terminus. These bacterial SCPs share approximately 20% sequence similarity with Urm1 in contrast to ubiquitin, which exhibits only weak similarity with Urm1. However, Urm1, UBLs, and SCPs share a characteristic diglycine motif at their C-terminus, which ensures their recognition for activation (see Figure 1A). Despite the low similarity of the primary sequence, these proteins all share a β-grasp fold, consisting of five β-sheets arranged around a central helix (see Figure 1B) [3]. In evolutionary terms, it is thought that UBL and SCP may have emerged from a single, multifunctional ancestral system capable of both adenylating and thiolating activities. Moreover, UBLs may have subsequently acquired the ability for protein conjugation activity; whereas SCP, may have further specialized with sulfur-transfer reactions [3,4]. Urm1 possesses both sequence and structural features of the last common ancestor; therefore, Urm1 is proposed to be a molecular fossil [3]. Consequently, this has allowed us to use Urm1 as a model to study the specialization of a UBL on tRNA thio modification at the expense of the lack of protein conjugation [3,4].

The Urm1-Uba4-system is not only restricted to eukaryotes, but also is found in archaeal systems. Ubiquitin-like SAMPs (small archaeal modifier proteins) were first identified in *Haloferax volcanii* [8,9,10,11]. The SAMP1, 2, and 3 are conjugated to their protein targets by an isopeptide bond. The SAMP-activating enzyme UbaA is critical for protein conjugation as well as sulfur mobilization during tRNA thiolation and molybdopterin biosynthesis.

In this review, we summarize recent insights into the role of Urm1 as the sulfur donor in tRNA thio-modification and compare the unique non-canonical UBL features of Urm1 to other UBLs and SCPs.

## 2. The Urm1 Pathway

**Figure 2:**
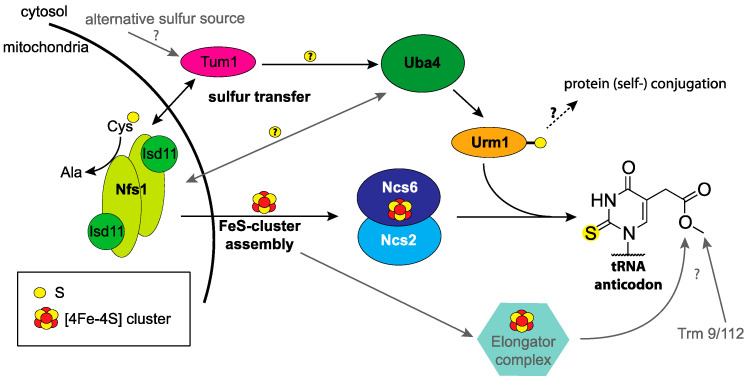
Biosynthetic pathway of the mcm^5^s^2^U modification. The schematic representation of the Urm1 pathway focuses on the sulfur flow (displayed as yellow balls) inside a yeast cell. Only the core components are displayed for clarity. For details see main text.

All tRNAs are decorated with nucleotides that are post-transcriptionally modified. These chemical modifications affect all aspects of tRNA processing, folding, and decoding. In particular, the anticodon loop is known to carry a large variety of chemical moieties [12,13]. Urm1 functions as the sulfur donor during 2-thiolation of the tRNA isoacceptors tE^UUC^, tQ^UUG^, and tK^UUU^ in all eukaryotes and tR^UCU^ in vertebrates [14,15]. This subset of tRNAs carry a 2-thio (s^2^) group on the wobble uridine (U_34_) and a methoxycarbonylmethyl group at position 5 (mcm^5^), leading to mcm^5^s^2^U_34_. Eight additional isoacceptors carry mcm^5^U or 5-carbamoylmethyl uridine (ncm^5^U), which are added by the elongator (Elp) complex and a protein complex consisting of the two tRNA methyltransferases Trm9 and Trm112 (Figure 2) [14,16,17,18].

The Urm1 pathway consists of four cytosolic proteins essential for 2-thiolation and several effector proteins [19]. The mitochondrial cysteine desulfurase Nfs1 (NiFS-like 1) and its stabilizing interaction partner Isd11 (iron-sulfur protein biogenesis, desulfurase-interacting protein 11) mobilize sulfur by converting cysteine into alanine. During this step, a persulfide forms on a catalytic cysteine of Nfs1 (Figure 2) [20]. Nfs1 is the only known cysteine desulfurase in *S. cerevisiae* that provides sulfur not only for tRNA thio-modification, but also for biosynthetic pathways that require sulfur including the iron-sulfur (FeS) cluster assembly machinery (see Figure 2) [21,22]. It was proposed that Tum1 (thiouridine modification protein 1) accepts sulfur from Nfs1 and shuttles between mitochondria and cytoplasm, where the sulfur is transferred onto Uba4 [23]. The same dual localization and an interaction with Uba4 have been established for MPST (mercaptopyruvate sulfurtransferase), which is the human Tum1 homolog [24,25]. Alternatively, a small fraction of Nfs1 may be present in the cytosol, since in vitro studies have shown that the Nfs1/Isd11 complex can facilitate persulfide formation on the RHD of Uba4 [26,27]. Uba4 activates the C-terminus of Urm1 by adenylation and subsequently generates the Urm1 thiocarboxylate [23,28,29]. This key step of the Urm1 pathway combines the characteristics of the UBL and SCP pathways (for details, see below). Following its thiocarboxylation, Urm1 acts as sulfur donor. As the final step, a protein complex consisting of Ncs2 and Ncs6 (needs *CLA4* to Survive 2 and 6) installs the sulfur on U_34_ [23,28]. Interestingly, Ncs6 contains a FeS-cluster critical for s^2^U formation [30,31,32]. Therefore, 2-thiolation requires Nfs1 through two independent pathways: first, its direct role for sulfur mobilization and second, through its role in FeS-cluster formation. Even though Nfs1 had been shown to be essential for 2-thiolation before, its dual role had not allowed to exclude indirect effects [33].

The Urm1 pathway is not the only pathway known to thiolate RNA nucleotides. However, the other known tRNA thio-modification pathways differ fundamentally from the Urm1-Uba4 system with respect to sulfur chemistry and the position of the thio-modified nucleotide. For example, in bacteria ThiI catalyzes the formation of s^4^U_8_, which is a critical sensor for UV light. The biosynthesis does not require an activating enzyme and the sulfur is installed via a sulfur-relay mechanism [34,35,36,37].

## 3. Cellular Phenotypes

*URM1* is a nonessential gene in yeast. However, initial studies revealed that *urm1*∆ yeast grows slightly slower than the wild type at 30 °C [2]. This changes dramatically at elevated temperatures (37 °C), where almost no growth of the mutant was observed [2]. This finding is in line with the observation that members of the Urm1 pathway are unstable at higher temperature, which directly affects the availability of modified tRNAs for translation [38,39,40]. Furthermore, the absence of any of the Urm1-pathway members leads to changes in morphology and growth characteristics. Similarly, these mutants are unable to invade media plates or to form pseudohyphal-filaments [41]. Interestingly, *URM1*-deficient yeast showed increased sensitivity to rapamycin, an inhibitor of TOR signaling [41], and oxidative stress induced by *tert*-butyl hydroperoxide (tBOOH) and diamide [42].

In mammalian systems, the homolog of Uba4, MOCS3 (molybdenum cofactor synthesis 3) not only thiocarboxylates URM1, but also MOCS2A, which is the sulfur donor in the molybdopterin-biosynthesis pathway [43,44]. Molybdenum-cofactor (Moco)-containing enzymes are critical for catalyzing a variety of redox reactions [45,46]. MOCS2A is similar to URM1 in primary sequence and structure as both contain a C-terminal diglycine motif that consists of a β-grasp fold. It has been shown that in yeast, Uba4 is capable to adenylate and thiocarboxylate human URM1 and MOCS2A in vitro [29]. In archaea, UbaA is required for anaerobic growth in the presence of DMSO, which serves as the terminal electron acceptor. This is explained by the requirement of the Moco for DMSO respiration [10,11]. In contrast, the Moco-biosynthesis pathway does not exist in baker’s yeast. This makes yeast an ideal model organism for studying the Urm1 pathway, since there is no need to discern phenotypes triggered by the absence of Moco synthesis when analyzing Urm1-pathway-related phenotypes.

Interestingly, in yeast all phenotypes that have been described in the absence of Urm1 function can be reversed by overexpressing the three tRNAs that are mcm^5^s^2^U_34_ modified in the wild-type cell [28,47,48]. This indicates that the observed effects are mainly caused by defects in tRNA modification rather than in Urm1 protein conjugation (see also Section 6).

## 4. The Urm1-Uba4 System

After the discovery of Urm1 through its homology to SCPs, Uba4 was identified in a yeast-two-hybrid screen [2]. Uba4 exhibits high sequence similarity with the adenylation domain (AD) of the ubiquitin activating enzyme Uba1 [2]. After overexpression of a tagged Urm1 protein or using polyclonal antibodies directed against Urm1, Western blot analyses revealed a pattern of high-molecular-weight species. This signal at least partially depended on the characteristic diglycine motif of Urm1 and on the function of Uba4. This has led to the classification of Urm1 as a ubiquitin-like protein modifier.

**Figure 3:**
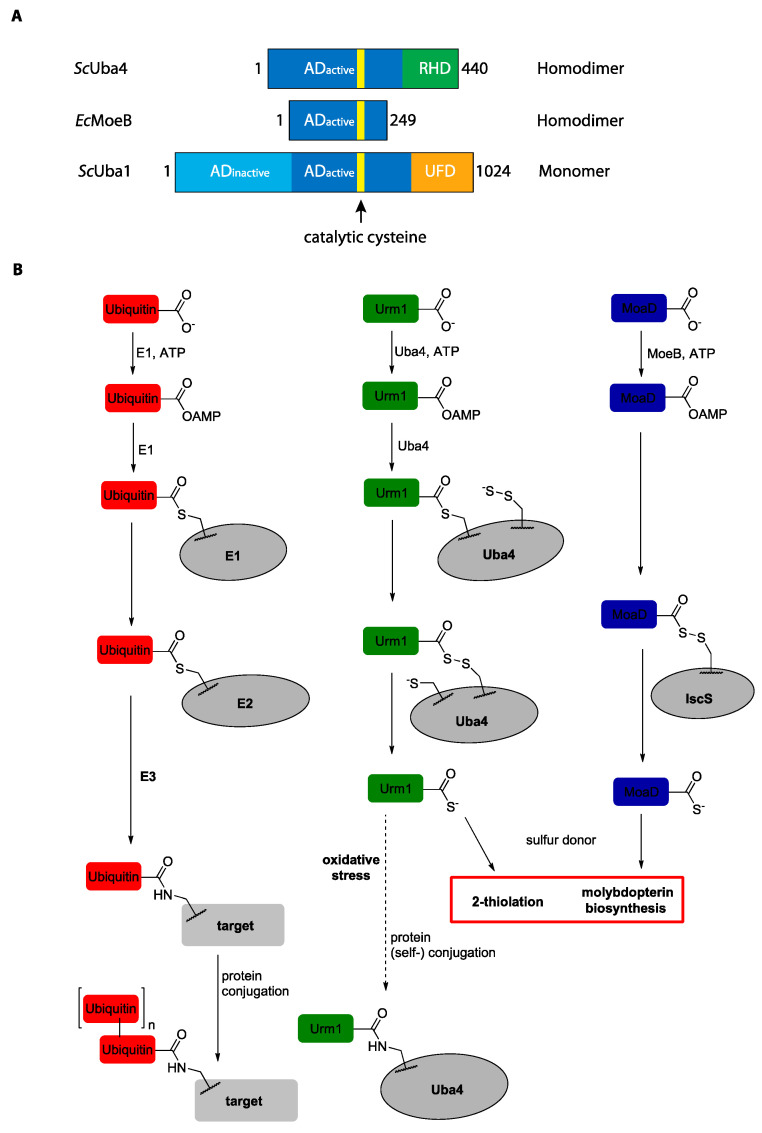
Domain overview of activating enzymes and mechanistical comparison of the Urm1-, ubiquitin- and MoaD-pathways. (**A**) Schematic representation of the Uba4 and Uba1 from *S. cerevisiae* and MoeB from *E. coli* [4,54,55]. The active adenylation domain is displayed in blue, whereas the inactive domain is shown in cyan. The rhodanese homology domain (RHD) and the ubiquitin fold domain (UFD) are colored in green and orange, respectively. The proteins are aligned according to the catalytic cysteine of the AD (yellow). Domain boundaries are indicated as well as the type of quaternary structure. (**B**) General mechanism of ubiquitin protein conjugation (left), the Urm1-Uba4 interaction (middle), and MoaD activation (right) (adopted from [49]). For details see main text.

Uba4 and its mammalian homolog MOCS3 are non-canonical E1-like activating enzymes and consist of two catalytically active domains: (i) an AD and (ii) a rhodanese homology domain (RHD) (Figure 3A). The AD is a common feature that Uba4 shares with all other E1 enzymes, the archaeal UbaA, and with MoeB and ThiF which are the bacterial activators of the SCPs. Canonical E1s like Uba1 contain an additional but inactive AD, which is neither found in Uba4 nor its bacterial counterparts. MoeB and Uba4 further differ from classical E1s by the absence of a ubiquitin fold domain (UFD) (Figure 3A). All E1 enzymes use an identical mechanism to activate their respective UBL by adenylating its C-terminal diglycine motif (Figure 3B). Following this initial step, the different systems diverge. This is indicated by the different intermediates and products which are formed. Subsequent to its adenylation, the C-terminus of Urm1 is further activated by forming a thioester with the catalytic cysteine of the AD of Uba4 [49]. Chemically, thioesters are high-energy bonds, which are much more susceptible to nucleophilic attacks than their oxyester counterpart like adenylates because thiols constitute better leaving groups [50]. The formation of a thioester is the central step in the classical UBL cascade, which is a tightly controlled process [51,52,53].

The catalytic cysteine of the AD is conserved between UBLs and SCPs (Figure 3A) [54]. However, a thioester intermediate between MoaD and MoeB has never been identified, suggesting that this mechanism may not be relevant in classical SCPs (Figure 3B) [56]. The RHD of Uba4 is a sulfurtransferase domain and is neither found in other E1 enzymes nor in activating enzymes of bacterial SCPs like MoeB (Figure 3A) [54,57]. In *E. coli*, the cysteine desulfurase IscS directly transfers the sulfur onto MoaD [58]. In the thiamin biosynthesis pathway, ThiF is required to mediate this process [59]. The cooperation of an activating domain with a sulfurtransferase domain is a unique feature and places the Urm1-Uba4-system at the evolutionary intersection of UBLs and SCPs.

The covalent thioester linkage between the C-terminus of Urm1 and the catalytic cysteine in the AD of Uba4 positions the thioester in close proximity of the RHD. This allows for a targeted nucleophilic attack of a persulfide that is present on the catalytic cysteine of the RHD, concomitantly cleaving the thioester and forming an acyl-persulfide bond [49,60]. The reductive cleavage of this bond leads to the Urm1 thiocarboxylate, which is a stable product (Figure 3B). An acyl-persulfide intermediate is characteristic for the SCPs. However, its formation in bacteria requires two distinct enzymes in contrast to the Urm1 pathway, where Uba4 harbors both functions. This mechanism is in stark contrast to canonical UBL-E1 systems, where a transthioesterification to an E2 and E3 ubiquitin ligase occurs to ensure the specificity of UBL conjugation. The high level of regulation of this step is reflected by the presence of more than 600 E3 ubiquitin ligases in human [61]. It is worth mentioning that the RHD is not a specificity module similar to E2 and E3 enzymes because the RHD adds a different enzymatic activity and does not provide specificity towards a particular protein target. The absence of E2 and E3 proteins in the Urm1-Uba4 system clearly distinguishes it from classical UBL and underlines Urm1′s function as a SCP. It has been proposed that the RHD may function as a built-in E2 domain. However, since the underlying chemistry is not identical, this concept has been abandoned [62,63]. The RHD ensures a specific reaction between the Urm1-thioester and persulfide, preventing cross-reactivity of the highly reactive intermediates with other cellular proteins. Importantly, the formation of an acyl-persulfide is the key difference to classical UBL systems, where a UBL forms an isopeptide bond with its target proteins. The final step of the Urm1-Uba4 reaction cascade is identical to the SCPs, where a cleavage of the central intermediate acyl-persulfide yields the thiocarboxylate.

Recently, the structure of full-length Uba4 was determined (PDB entry 6YUB) [64]. Surprisingly, Uba4 forms an asymmetric homodimer, where both RHD interact with only one AD. It was already known that Uba4 forms a homodimer and that it can form a complex with Urm1, human URM1, or MOCS2A [28,29]. The new structure established that the catalytic cysteine of the RHD is buried within the RHD interface, therefore, not accessible. To induce a conformational rearrangement within Uba4, the binding of Urm1 and ATP are required. First, Urm1 gets adenylated and subsequently, a thioester is formed in order to generate the functional relevant thiocarboxylated Urm1 [23,28,49]. This stepwise process and in particular the movement of the linker between the two domains is tightly controlled [64]. It has been shown that a CxxC motif in this region is important to control this process in other E1 enzymes [65]. The catalytic cysteine (C225 in baker’s yeast) of the AD is located in the crossover loop, a flexible loop region that is positioned to facilitate a nucleophilic attack of the adenylated Urm1 to form a thioester [49]. This feature of Urm1 has recently been exploited to trap the transition state and to gain structural insights into the activation mechanism of Urm1 [64]. To achieve this, the catalytic cysteine was mutated to lysine (C202K) to form a stable isopeptide bond between the Urm1 C-terminus and Uba4.

The cocrystal structure of Uba4-K202-Urm1 (PDB entry 6YUC) provides structural insights into the catalytic cascade by revealing that Urm1 is bound to Uba4 in a transition state. Since the position of the RHD could not be determined in this structure, further analyses will be required to complete the picture of the full reaction cycle and, in particular, the sulfur transfer.

## 5. Urm1 Conjugation

Urm1 and Uba4 were initially described as a protein-conjugation system [2]. According to these findings, Urm1 can be conjugated to several target proteins in an Uba4-dependent process called urmylation [41]. Further investigations led to the identification of Ahp1 (alkyl hydroperoxide reductase 1) as a target protein [42]. Ahp1 acts as an antioxidant by reducing organic hydroperoxides, hence protecting cells from oxidative damage [66]. A series of chemical probing experiments led to the conclusion that Urm1’s C-terminal thiocarboxylate is crucial for the attachment of Urm1 via an isopeptide bond [67]. A mechanistic dissection identified lysine 32 (K32) of Ahp1 as the residue for Urm1 conjugation [67]. Importantly, conjugates are exclusively formed upon oxidative stress. Therefore, the efficiency of conjugate formation is affected by the use of different oxidizing agents like diamide or H_2_O_2_, whereas, the overall conjugation pattern appears rather similar. Interestingly, Peroxiredoxin 5 (Prx5) the fly homolog of Ahp1 was found to be conjugated to Urm1 in the absence of oxidative stress [68]. On the contrary, the absence of Urm1 or its activating enzyme Uba4 confers increased resistance to the oxidative stressor paraquat when applied by food. This phenotype was explained by the upregulation of several signaling pathways, in particular the JNK-pathway [68]. An in-depth proteomic approach in *Drosophila melanogaster* subsequently led to the identification of additional targets. These targets can be grouped into functional networks for tRNA modification, redox-processes, cytoskeletion organization, RNA processing, translation, and protein folding [69]. Recently, Urm1 conjugation in the archaeon *Sulfolobus acidocaldarius* was linked to proteasomal degradation without mentioning the formation of Urm1-COSH or a function in tRNA thiolation [70]. It is noteworthy that a comparison to archaeal homologs is not straight-forward since their activating enzymes do not contain a RHD [70]. Besides several unrelated target proteins, pathway members of the Urm1 pathway like Uba4, Ncs2, and Ncs6 were conjugated to Urm1 upon oxidative stress in humans and flies [67,69].

Despite the early discovery of Urm1 protein conjugates, the physiological role of the Urm1 protein conjugation remains enigmatic. The high reactivity of thiocarboxylated Urm1, the presence of reactive sulfur intermediates, and the lack of E2 and E3 enzymes make it likely that the phenomenon is mainly a side reaction. Thiolates and thiocarboxylates are susceptible to oxidation due to their nucleophilic nature [71,72,73]. Furthermore, oxidized intermediates like acyl persulfides are prone to nucleophilic attacks and form stable isopeptide bonds with lysines in their close proximity because thiols and persulfides are excellent leaving groups [50,71]. The requirement of a thiocarboxylate as the reactive group is the major chemical difference to classical UBL. There, enzymatic cascades lead to specific conjugation in a spatially and temporally defined manner; whereas, Urm1 conjugation is not subjected to similar control mechanisms due to the lack of dedicated E2/E3 enzymes. It is possible that the observed conjugation pattern is merely the consequence of an environmental stimulus like oxidative stress that alters the cellular metabolism and not a physiologically directed process linked to biological function. Current studies seek to better characterize the critical parameters and conditions that underlie and regulate the formation of these Urm1-conjugates [74]. To support the physiological relevance of urmylation, it will be critical to identify all reaction intermediates and chemical species which lead to Urm1 conjugation.

To uncover the function of the Urm1 pathway, chemical probes have been used. For instance, an electrophilic vinyl methyl ester at the C-terminus of Urm1 was utilized to identify interaction partners [75]. This method has previously been applied to ubiquitin to identify catalytic cysteines, in particular of deubiquitinases (DUBs) [76]. It is noteworthy that neither known members of UBL conjugation machineries nor deubiquitinating enzymes were identified in this study [75]. This poses the questions whether Urm1 conjugation is specific and regulated similar to UBL. Furthermore, recent in vitro studies have characterized the mechanism of Urm1-conjugate formation in more detail [64]. However, while identifying a self-protective mechanism of Uba4 to reduce Urm1 conjugation, these experiments did not address the in vivo role of Urm1 conjugation [64]. It is challenging to discriminate directed Urm1 conjugation from nonspecific side reactions, which occur in close proximity to the reactive species [64]. To unambiguously show a physiological effect of urmylation we will need to identify a phenotype which is independent of 2-thiolation. Currently, such a phenotype is not known. Hence, it is most likely that the known phenotypes are triggered by alterations of tRNAs modifications and the downstream change of cellular translational programs. Therefore, we lack the evidence that Urm1 conjugation is used to trigger a specific cellular response. Sophisticated in vivo studies will be required to interconnect Urm1 protein conjugation, the role of environmental stimuli, tRNA thiolation levels, and translational programs. However, it will be challenging to experimentally separate these different aspects for a comprehensive interpretation.

## 6. Molecular Phenotypes and Implications for Higher Organisms

The absence of Urm1 function results in complex phenotypes, which blend direct and indirect effects (see Section 3). However, what are the underlying molecular mechanisms?

The molecular target of Urm1-mediated thiolation are nucleosides in the anticodon of a small subset of tRNA isoacceptors [23,28]. This has led several groups to focus on the mechanistic role of 2-thiolation during mRNA translation. Importantly, phenotypes of Urm1 deficiency can be suppressed, similar to mutants of the Elp complex [47], by overexpressing the normally mcm^5^s^2^U-modified tRNA. In contrast, overexpressing the near-cognate isoacceptors fails to rescue the phenotypes, showing that the effect is codon specific [28,48,77]. This finding was further supported by a translation reporter that revealed an increase of frameshifting events and amino acid misincorporation in tRNA-modification mutants [78]. Furthermore, ribosome profiling was used to generate high-resolution snapshots of in vivo translation dynamics [79], showing that AAA and CAA codons are decoded more slowly in mcm^5^s^2^U-deficient yeast [77,80,81]. This codon-specific slowdown is linked to defects in protein homeostasis and the aggregation of misfolded proteins in yeast [77,82,83]. In addition, Ranjan and colleagues performed in vitro translation experiments using tRNA purified from wild type, *urm1*Δ and *elp3*Δ yeast. They found that A-site binding of hypomodified tRNA was impaired due to decreased tRNA association (k_on_) and increased tRNA dissociation (k_off_) during decoding of the cognate codon in comparison to the fully modified isoacceptor. This effect also led to an increased rate of rejection of unmodified tRNA during A-site decoding [84,85]. Finally, the lack of s^2^U leads to decreased efficiency of ribosomal rearrangement and slower tRNA-mRNA translocation [85], which is in line with the ribosome profiling experiments. This emphasizes that seemingly subtle perturbations of translation dynamics may underlie complex cellular phenotypes.

The observed protein-homeostasis defect likely explains the reduced viability in yeast and nematodes [77]. The fact that tRNA overexpression is sufficient to suppress codon-specific translational slowdown, protein aggregation, cellular phenotypes, and to restore gene expression indicates that the observed slowdown in mutants is the underlying reason [77]. The cellular phenotype is, therefore, a systemic defect and unlikely to target a specific pathway [86,87]. Nevertheless, the absence of Urm1 function was linked to Tor signaling through multiple observations. First, mutants of the Urm1-pathway are sensitive towards the Tor inhibitor rapamycin. Second, genetic analyses have shown that *urm1*Δ can be rescued by expressing a constitutively active allele of *TOR2* [41]. Third, the absence of Uba4 and Urm1 induces an uncoupling of nutrient availability and transcription-factor control of amino acid biosynthesis [88,89]. However, in this study the role of Urm1 protein conjugation and its role as sulfur donor in tRNA modification were not investigated. Finally, even though mutants of the Urm1 pathway have not been studied by metabolomics in detail, the analysis of *ELP* mutants revealed an altered metabolomic profile [90]. The central role of the Tor kinase for the integration of nutrient signaling, cellular metabolism, and stress responses, explains why this pathway is particularly triggered like in other general stress responses [91]. This link may also explain the reduced ability of the mutants for invasive growth since Tor-mediated glucose signaling is known to trigger invasive growth [41].

In archaea, similar to yeast, the lack of the SAMP-activating enzyme UbaA or SAMPs leads to a complete loss of thiolated tRNA [10,11]. An initial analysis of Urm1 in human cells, has reported that the downregulation of Urm1 induces cytokinesis and cell-cycle defects [75]. This is consistent with findings in yeast, where the loss of different tRNA-modifying enzymes leads to similar effects [82,92]. However, the analysis of a recently generated full knockout of *MOCS3* is more complex, since molybdopterin biosynthesis and tRNA thiolation are both affected in these cells [93].

These findings emphasize the physiological relevance of the Urm1 pathway across species as well as the role of chemical tRNA modifications. Defects in their regulation are associated with multiple neurological disorders and several types of cancer [94,95]. While the full knockout of an Elp-pathway member is embryonic lethal in mice [96] a conditional knockout of Elp3 in the developing cortex of mice impairs neurogenesis by triggering the unfolded protein response (UPR) in the endoplasmatic reticulum (ER) [97]. The perturbation in protein homeostasis and cellular signaling was shown to alter the differentiation patterns of neuronal progenitors, resulting in microcephaly [97]. Furthermore, in the hereditary disease familial dysautonomia (FD), a splicing defect in a subunit of the human Elp-complex leads to decreased levels of mcm^5^s^2^U [98,99,100]. FD patients experience neuro-developmental defects that result in the loss of sensory neurons and are associated with severe cardiovascular symptoms, a lack of the ability to feel pain, and premature death [101]. The splicing defect as well as the lack of mcm^5^s^2^U can be corrected in cell culture by the addition of the small compound RECTAS, revealing a potential treatment option [15]. Similarly, deletion or mutations of Urm1-pathway members can lead to severe effects. For example, an altered splicing variant of *NCS2* causes the DREAM-PL syndrome, which is characterized by dismorphic features and microcephaly [102].

All these pathologies are triggered by tRNA hypomodification. In cancer, however, elevated levels of tRNA modifying enzymes and tRNA modifications responsible for mcm^5^s^2^U synthesis contribute to the formation of malignant tumors and facilitate metastasis formation in breast cancer models and in melanoma [103,104]. This fits with observations in many tumors that are characterized by impaired translation control, leading to an increased protein synthesis [105]. Currently, only one study links Urm1 conjugation to a disease. According to this report, Urm1 is conjugated to the viral oncoprotein Tax in human and fly models, leading to Tax redistribution to the cytosol thereby promoting adult T-cell leukemia (ATL). Interestingly, the localization of Tax is also controlled by ubiquitin and SUMO [106].

In summary, the evidence that implicates the Urm1 pathway in disease, renders it very promising to establish the Urm1-Uba4-system as a novel drug target to inhibit translational processes in cancer [107,108]. The E1 enzymes of NEDD8 and SUMO have already been targeted in human malignancies [109,110]. The use of these mechanistic inhibitors targets the UBL-E1-thioester intermediates thereby promoting a specific inhibition. Since the structural basis of the specificity has been revealed [111], the recent structure of the thioester-mimic between Urm1 and Uba4 may act as a suitable starting point to specifically target the complex by similar inhibitors [64].

## 7. Outlook

The scaffold of many tRNA modifications consists of a metabolite [13]. Therefore, it is tempting to speculate that the cellular sulfur metabolism or redox state might directly affect 2-thiolation levels, leading to a change of translational programs. The connection between the availability of sulfur-containing amino acids (cysteine and methionine) and the incorporation of 2-thiouridine has already been made. According to this model, limited nutrient availability affects amino acid biosynthesis and the metabolism of sugars and carbon [89,112,113]. For a deeper understanding, it will be necessary to identify the direct sulfur donor of Uba4. Nfs1 is the sole cellular cysteine desulfurase, suggesting a direct route to cysteine. However, careful investigations will be needed to exclude indirect effect by other sulfur-related pathways like FeS-cluster assembly. The impairment of FeS-cluster maturation would disrupt the catalytic activity of different tRNA modifying enzymes like Ncs6 for s^2^U and Elp3 for mcm^5^ and ncm^5^. Recent studies have revealed that a fraction of Nfs1 localizes to the cytosol [25,93]. This finding necessitates a new interpretation of the role of Tum1, which was thought to act as shuttle between mitochondria and cytosol [23]. In the absence of Tum1, residual levels of 2-thiolation still occur [23,28]. However, cells might simply use multiple pathways for sulfur supply. The identification of the key metabolites would allow for an in-depth analysis of the interplay between metabolism and translation. Tum1 is capable of utilizing 3-mercaptopyruvate as its sulfur source, offering a potential link to other metabolic processes like the cysteine metabolism [24]. However, it is worth noting that the thiolated tRNA pool is very stable and exhibits a low turnover rate, which buffers changes of 2-thiolation rates [40]. Hence, mRNA translation may react to changes in s^2^U levels relatively slowly and not likely as a fast switch for gene expression [114].

Until now, Urm1 protein conjugation has been found under rather artificial conditions like overexpression, pull-down, and in vitro assays. This has prevented a clear link to a physiological function. Furthermore, Urm1 conjugation is predominantly observed in the presence of an oxidative stressor. It is known that Ahp1 is able to inactivate small organic peroxides, thereby protecting cells from oxidative damage. This raises the question whether the thiocarboxylated C-terminus of Urm1 might be misrecognized as an Ahp1-substrate subsequently leading to conjugation of the two proteins. It is possible, that Urm1 conjugation through a thiocarboxylate is the remnant of an ancestral protein-conjugation system. Since a thiocarboxylate is a highly reactive nucleophile, this strategy poses a considerable risk to the cell. By fusing the AD and RHD in a single enzyme, it ensures that the sulfur is specifically installed and shielded from a cross reaction with other components of the cell. The eukaryotic Urm1-Uba4 system appears to be more sophisticated when compared to their bacterial counterpart MoeB-MoaD. This might be explained by the requirement to provide sulfur for two different processes, tRNA thiolation and Moco biosynthesis in most eukaryotes. However, species like *S. cerevisiae* have maintained the Urm1-Uba4 system despite the absence of proteins that use the Moco. This might be a sign for improved functionality of Uba4, even though it cannot be excluded that Urm1 serves an additional, yet undiscovered function.

So far, no proteolytic activity has been identified to reverse Urm1 protein conjugation. This is in contrast to canonical UBLs, where specific DUBs play an important role in controlling protein conjugation. Furthermore, nothing is known about the dynamic of this process or the stability of Urm1 conjugates. The absence of such enzymatic activity further indicates that the key function of the Urm1-Uba4 system is primarily and maybe exclusively to act in sulfur transfer and not in protein conjugation. This raises the question whether Urm1 protein conjugation plays a functional role or is merely a remnant of ancient UBL- and SCP evolution, where Urm1 has kept an intrinsic and basal feature of protein conjugation during its evolution.

Hence, 20 years after its discovery, the Urm1-Uba4 system has still not revealed all its secrets. However, its unique position in the evolutionary tree of UBL and SCP, offers unique opportunities. Urm1 may provide a window to study UBL evolution highlighting a time when protein conjugation occurred as a byproduct of SCP activity before the cell invented current sophisticated regulatory mechanisms that have thrust UBL into the limelight of cellular regulation.

## Data Availability

Data sharing is not applicable.
